# Novel *MYH7* Variants in Indian Patients With Hypertrophic Cardiomyopathy

**DOI:** 10.1016/j.jacasi.2026.08.028

**Published:** 2026-11-03

**Authors:** Deepa Selvi Rani, Pratibha Nallari, Calambur Narasimhan, Kumarasamy Thangaraj

**Affiliations:** a CSIR-Centre for Cellular and Molecular Biology, Hyderabad, India; b Department of Genetics, Osmania University, Hyderabad, India; c Department of Cardiology, AIG Hospitals, Hyderabad, India

**Keywords:** 3D protein structure, hypertrophic cardiomyopathy (HCM), root mean square deviation (RMSD), sarcomere genes, β-myosin heavy chain gene (β-MYH7)

Hypertrophic cardiomyopathy (HCM) is an inherited myocardial disorder characterized by unexplained left ventricular hypertrophy, impaired diastolic function, ventricular arrhythmias, heart failure, and sudden cardiac death.^1^^,^^2^ Pathogenic variants in the *MYH7* gene, which encodes the β-myosin heavy chain, are a major genetic cause of HCM.^1^, ^2^, ^3^, ^4^, ^5^, ^6^ However, the pathogenic significance of rare *MYH7* variants in Asian populations remains incompletely understood. To address this knowledge gap, we analyzed the spectrum and pathogenicity of *MYH7* variants in Indian patients with HCM.

We studied 160 unrelated patients with clinically diagnosed HCM (mean age: 55 ± 12 years; 113/160 [70.6%] man) and 178 ethnically matched healthy controls (mean age: 56 ± 12 years; 123/178 [69.1%] man). Patients had a mean interventricular septal thickness of 23.2 ± 4.2 mm, a mean left ventricular ejection fraction (LVEF) of 49% ± 8%, a family history of HCM in 115/160 (71.9%), and a family history of sudden cardiac death in 33/160 (20.6%). The relatively broad range of LVEF reflects the clinical heterogeneity of the study group. The study was approved by the Institutional Ethics Committees of the CSIR (Council of Scientific and Industrial Research)-Center for Cellular and Molecular Biology and the Department of Genetics, Osmania University, Hyderabad, India, and written informed consent was obtained from all participants. Principal component analysis using 47 ancestry-informative markers in the present (160 HCM/178 controls) and previous South Indian cohorts (209 HCM/230 controls) showed substantial overlap between cases and controls; the HapMap CEU and YRI reference study group formed distinct clusters, supporting comparable genetic ancestry between the case and control groups.

Genomic DNA was isolated from peripheral blood samples, and all coding exons together with exon–intron boundaries of *MYH7* were analyzed by bidirectional Sanger sequencing. Variants were interpreted using clinical phenotype, family segregation, gnomAD frequency, ClinVar, functional-domain localization, PolyPhen-2, Sorting Intolerant from Tolerant, and American College of Medical Genetics and Genomics (ACMG)/Association for Molecular Pathology (AMP) criteria.^1^^,^^4^ Homology models based on human β-cardiac myosin motor domain (PDB ID: 4P7H) were used to assess structural effects.^7^

Our study identified 34 *MYH7* variants, comprising 10 missense (p.Met362Leu, p.Lys367Asn, p.Gly389Lys, p.Ala423Thr, p.Val431Met, p.Glu525Lys, p.Met684Arg, p.Arg723His, p.Leu725Pro, and p.Asp896Asn) (Figure 1A), 2 splice-site, 2 frameshift and remaining are synonymous and intronic variants. All novel variants were submitted to dbSNP (Batch ID: 1062022). Among the missense variants, p.Met362Leu was recorded as benign, whereas p.Lys367Asn and p.Gly389Lys were classified as variants of uncertain significance. Attempts were made to obtain samples from family members for segregation analysis of p.Lys367Asn and p.Gly389Lys; however, family samples were unavailable. The 5 missense variants p.Ala423Thr, p.Val431Met, p.Glu525Lys, p.Arg723His, and p.Asp896Asn, identified in the present cohort, had previously been reported by our group in independent South Indian HCM cohorts.^5^^,^^6^ Interestingly, 2 novel variants, p.Met684Arg and p.Leu725Pro were absent from gnomAD, 1000 Genomes, ESP, ExAC, and the healthy control cohort supporting their rarity. Furthermore, both were located within highly conserved and functionally important regions of the myosin motor domain, suggesting potential functional significance. Together with the clinical, segregation, computational, and structural evidence, these findings supported their classification as likely pathogenic according to ACMG/AMP criteria.^1^, ^2^, ^3^, ^4^

**Figure 1:**
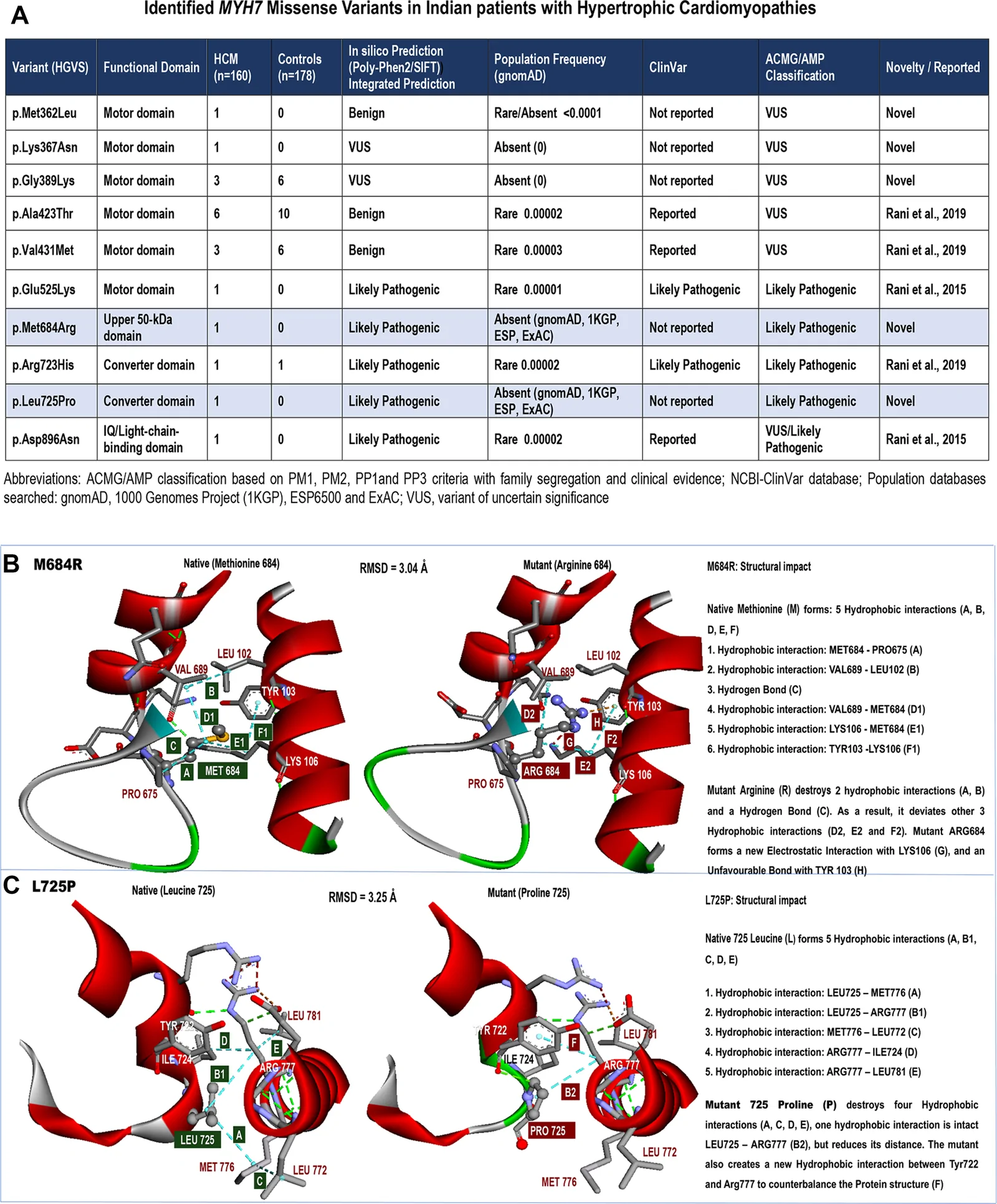
Genetic and Structural Characterization of *MYH7* Variants in Indian HCM (A) Identified *MYH7* missense variants in Indian patients with hypertrophic cardiomyopathy and analyzed them according to functional domain, case-control occurrence, in silico prediction, population frequency, ClinVar status, and ACMG/AMP classification. p.Met684Arg and p.Leu725Pro were absent from gnomAD, 1000 Genomes (1KGP), ESP, ExAC, and the healthy control cohort. (B) Structural comparison of native and mutant p.Met684Arg (M684R), showing changes in local molecular interactions and a root mean square deviation (RMSD) of 3.04 Å. (C) Structural comparison of native and mutant p.Leu725Pro (L725P), showing changes in local molecular interactions and an RMSD of 3.25 Å. ACMG/AMP = American College of Medical Genetics and Genomics/Association for Molecular Pathology.

The p.Met684Arg was identified in a 52-year-old woman with symptomatic HCM, severe asymmetric septal hypertrophy (25 mm), preserved LVEF (55%), and NYHA functional class III symptoms. Her affected 26-year-old son (first-degree relative) carried the variant and had mild left ventricular hypertrophy, providing limited supportive segregation evidence and suggesting possible age-dependent phenotypic expression. The p.Met684Arg variant is located within the upper 50-kDa motor domain, a functionally important region that contributes to actin binding and is structurally coupled to the adenosine triphosphate hydrolysis active site, thereby facilitating force generation during the myosin mechanochemical cycle.

p.Leu725Pro was identified in a 58-year-old man with hypertrophic obstructive cardiomyopathy, asymmetric septal hypertrophy (23.4 mm), preserved LVEF (56%), NYHA functional class III symptoms, and a family history of sudden cardiac death. His affected 24-year-old daughter (first-degree relative) carried the variant and had mild HCM, providing limited supportive segregation evidence. The p.Leu725Pro variant is located within the converter domain adjacent to the lever arm, a region that is essential for transducing adenosine triphosphate-driven conformational changes into lever-arm rotation (the power stroke) and contributes to stabilization of the interacting-heads motif in the relaxed state.

Structural modeling showed localized perturbations while preserving overall β-cardiac myosin architecture. Root mean square deviation values were 3.04 Å for p. Met684Arg and 3.25 Å for p. Leu725Pro; AlphaMissense scores were 0.99 for p. Met684Arg and 0.999 for p.Leu725Pro.^8^ Both substitutions altered native molecular interactions, and PolyPhen-2 and Sorting Intolerant from Tolerant predicted both variants to be deleterious. Structural findings were supportive rather than equivalent to experimental functional evidence, and root mean square deviation values were not treated as pathogenicity thresholds. The observed structural alterations are consistent with mechanisms reported for established pathogenic *MYH7* variants, including p.Arg403Gln (R403Q), p.Arg453Cys (R453C), and p.Arg719Trp (R719W), which have been associated with myocyte hypercontractility, altered intracellular calcium homeostasis, enhanced force generation, and destabilization of the interacting-heads motif despite preservation of the overall protein architecture.^3^

Rare *MYH7* variant frequencies were compared between HCM cases and controls using Fisher exact test because of the small cell counts. The novel variant was present in 1/160 HCM cases (0.63%) and 0/178 controls (*P* = 0.473). No multiple-testing correction was applied because this was a single formal comparison. The nonsignificant result does not independently establish pathogenicity, and the limited sample size and rarity of the variant reduced statistical power.

For p.Met684Arg, evidence supporting likely pathogenicity included absence from population databases and controls, limited supportive segregation, concordant computational predictions, and localization in a conserved, functionally important *MYH7* motor-domain region. For p.Leu725Pro, the same evidence categories applied, including localization in a conserved functional region. The case-control observation of 1/160 vs 0/178 (*P* = 0.473) was not used as independent proof of pathogenicity. Rather, the absence of the variants from patient databases and controls, segregation with a first-degree relative having HCM, biologically important localization, and concordant computational and structural findings provide convergent support for a deleterious role. No functional assay was performed, no conflicting evidence was identified, and the limited pedigrees precluded meaningful quantitative segregation analysis or LOD scores. Overall, the integrated clinical, study group, segregation, computational, structural, and ACMG/AMP evidence supports classification of both variants as likely pathogenic.^9^^,^^10^

Accurate interpretation of rare *MYH7* variants is important for molecular diagnosis, cascade family screening, genetic counseling, and risk stratification. These findings expand the *MYH7* mutational spectrum in Indian patients with HCM and contribute data from an underrepresented study group.

## Author Contributions

D.S.R., P.N., and K.T. conceived and designed the experiments. D.S.R. performed the experiments and wrote the manuscript. P.N. and C.N. contributed clinical samples and control samples. K.T. provided critical suggestions.

## Funding Support and Author Disclosures

Dr Rani was supported by the Council of Scientific and Industrial Research (CSIR). Dr Thangaraj was supported by the CSIR Bhatnagar Fellowship (GDA No. 24 [P90807]), CSIR, Government of India. All other authors have reported that they have no relationships relevant to the contents of this paper to disclose. The funders had no role in the study design, data collection, analysis, decision to publish, or article preparation.
